# A Comparison of Some Organizational Characteristics of the Mouse Central Retina and the Human Macula

**DOI:** 10.1371/journal.pone.0125631

**Published:** 2015-04-29

**Authors:** Stefanie Volland, Julian Esteve-Rudd, Juyea Hoo, Claudine Yee, David S. Williams

**Affiliations:** Departments of Ophthalmology and Neurobiology, Stein Eye Institute, Molecular Biology Institute, Brain Research Institute, David Geffen School of Medicine at UCLA, Los Angeles, CA, United States of America; National Eye Institute, UNITED STATES

## Abstract

Mouse models have greatly assisted our understanding of retinal degenerations. However, the mouse retina does not have a macula, leading to the question of whether the mouse is a relevant model for macular degeneration. In the present study, a quantitative comparison between the organization of the central mouse retina and the human macula was made, focusing on some structural characteristics that have been suggested to be important in predisposing the macula to stresses leading to degeneration: photoreceptor density, phagocytic load on the RPE, and the relative thinness of Bruch’s membrane. Light and electron microscopy measurements from retinas of two strains of mice, together with published data on human retinas, were used for calculations and subsequent comparisons. As in the human retina, the central region of the mouse retina possesses a higher photoreceptor cell density and a thinner Bruch’s membrane than in the periphery; however, the magnitudes of these periphery to center gradients are larger in the human. Of potentially greater relevance is the actual photoreceptor cell density, which is much greater in the mouse central retina than in the human macula, underlying a higher phagocytic load for the mouse RPE. Moreover, at eccentricities that correspond to the peripheral half of the human macula, the rod to cone ratio is similar between mouse and human. Hence, with respect to photoreceptor density and phagocytic load of the RPE, the central mouse retina models at least the more peripheral part of the macula, where macular degeneration is often first evident.

## Introduction

More so than for other neurodegenerations, the field of retinal degeneration has been assisted by numerous mouse models that have provided close parallels with the human disease. The first inherited form of retinal degeneration to be reported in a mouse strain occurred 90 years ago [1]. Since then, mouse models for a variety of monogenic inherited retinal degenerations have been identified by mouse geneticists or developed by genetic engineering [2, 3]. Extensive studies performed on these models have led to a wealth of understanding about the molecular pathogenesis underlying the retinal degenerations.

Macular retinal degenerations affect the central retina, and thus impair high acuity vision. Age-related macular degeneration (AMD) is a major cause of blindness amongst the elderly [4, 5]. AMD is a more difficult retinal disease to study due to its late onset, complex genetics and the involvement of environmental factors in its onset and progression. Mouse models exhibiting some of the features observed in AMD have been developed [6–9]. However, the mouse retina does not have a macula *per se*, leading to the question of whether the mouse is a relevant model for macular degeneration.

The macula subtends an angle of ~20 degrees in the center of human and many non-human primate retinas; the optic disc lies at the nasal edge of the macula. The macula has a number of characteristics that distinguish it from other parts of the retina. It has been referred to as the macula lutea, meaning yellow spot, due to the presence of the carotenoids, zeaxanthin and lutein [10]. The center of the macula consists of the fovea centralis, where cone photoreceptors are packed at their highest density. Surrounding the fovea, the macula contains rod photoreceptors, packed at a density that compares to that of the foveal cones [11–13].

Why the macula may be more susceptible to degeneration is not known. The concentration of zeaxanthin and lutein has been thought to be protective rather than a liability [14]. However, there are a variety of characteristics that are more likely to contribute to this susceptibility. Some of these characteristics are related to the structural organization of the macula, especially with respect to its retinal pigment epithelium (RPE), which has been suggested to be the initial site of pathogenesis in macular degeneration [15, 16].

One suggestion has been that differences in the thickness of Bruch’s membrane may contribute to this susceptibility [17]. Bruch’s membrane is the basement membrane of the RPE cells and thus provides a barrier between the RPE and choroidal capillaries. It has been shown to be thinner in the macula than in the rest of the human retina [17, 18]. In wet AMD, which corresponds to 10–15% of AMD cases, choroidal neovascularization that penetrates Bruch’s membrane promotes deterioration of the macula.

Another potential contributor to the vulnerability of the macula is its high density of photoreceptor cells, especially in relation to the high metabolic load this density places on the RPE cells, which must degrade the shed outer segment disk membranes of the photoreceptors [19]. For forty years, it has been proposed that inefficiencies in outer segment phagosome degradation lead to RPE pathogenesis, and age-related visual impairment, as in AMD [15, 20–24]. Undigested outer segment membranes are thought to contribute to deposits, such as drusen, lipofuscin, and basal laminar and linear deposits, and numerous reports suggest a link between these deposits and macular degeneration [25–33].

To address the question of the relevance of mouse models for macular degeneration, it is important to assess the mouse retina with respect to parameters that may be related to susceptibility to macular degeneration. Given the suggestions that rod and cone photoreceptor density, phagocytic load on the RPE, and the relative thinness of Bruch’s membrane may play a role in susceptibility to macular degeneration, in the present study, we have compared these structural characteristics between the mouse retina and the human macula.

## Materials and Methods

### Animals

In all experiments two WT strains of mice, C57BL/6J and BALB/C were used. Mice were kept at a 12 h light/12 h dark cycle and were sacrificed at an age of 8 weeks. These experiments were performed in accordance to the guidelines established by the Animal Research Committee of the University of California Los Angeles and the ARVO Statement for the Use of Animals in Ophthalmic and Vision Research.

### Microscopy of sectioned retinas

All chemicals were purchased from Fisher Scientific or Electron Microscopic Sciences. Eyes were enucleated and fixed overnight at 4°C in 2% formaldehyde, 2.5% glutaraldehyde in 0.1 M sodium cacodylate buffer pH 7.4. After removal of the anterior segment, eyecups were bisected along the dorsal ventral axis, and then postfixed in 1% osmium tetroxide in 0.1 M sodium cacodylate buffer for 1–2h, and dehydrated. One half of each eyecup was embedded in a mixture of EMbed-812 and Araldite 502 (Electron Microscopy Sciences; Hatfield, PA), and the other in Araldite 502. Semithin (~700 nm) sections were collected from the Embed-812/Araldite 502 blocks, stained with Toluidine blue and imaged with a Zeiss Axio Imager. Ultrathin sections were prepared from Araldite 502-embedded samples, stained with 5% uranyl acetate in ethanol and 0.4% lead citrate, and imaged with a Zeiss EM 910. Photoreceptor density was determined by counting the number of rod and cone cell nuclei in a series of 50-μm segments along dorso-ventral semithin sections, imaged with a 63x oil immersion objective. The density of the photoreceptor mosaic in an area of 2500 μm^2^ was thus calculated. Distance along the retinal arc was converted into eccentricity, based on a posterior nodal distance for the mouse eye of 1.75 mm [34]. Measurements of the thickness of the Bruch’s membrane, as well as the thickness of its elastin layer, were made from electron micrographs at a magnification of 5000 times, using ImageJ v1.43u software (developed by Wayne Rasband, National Institutes of Health, Bethesda, MD; available at http://rsb.info.nih.gov/ij/index.html). Data were collected from 4–8 different individuals for each mouse line.

### Microscopy of RPE Wholemounts

Eyecups were obtained from enucleated eyes in Ringer’s solution (130 mM NaCl, 3.6 mM KCl, 2.4 mM MgCl_2_, 1.2 mM CaCl_2_, 10 mM HEPES with KOH [pH 7.4], and 20 mMEDTA), and then 4 incisions from the periphery towards the optic nerve were made in order to flatten the eyecup. The sensory retina was gently removed with a brush, and the RPE and choroid were transferred to a wet nitrocellulose filter on a glass slide. The resulting wholemounts were oriented, fixed in 4% formaldehyde for 1h at RT, and washed in PBS and blocked with 1% BSA and 0.2% tween-20 in PBS for 1h at RT. They were then washed in PBS, incubated in FITC-phalloidin at 1 μg/ml (Sigma-Aldrich; St. Louis, MO) and DAPI at 2 μg/ml (Life Technologies; Carlsbad, CA) in 1% BSA, 0.2% tween-20 in PBS for 2h at RT, and finally washed in PBS and mounted on slides. They were imaged with an Olympus Fluoview FV1000 confocal laser microscope. The surface area of each RPE cell was determined using ImageJ.

### Statistical Analysis

Measurements of the different morphological parameters obtained from the eccentricities for each mouse strain were compared using a one-way ANOVA and Tukey’s *post-hoc* test, with *P* < 0.050 indicating statistically significant differences.

## Results

### Photoreceptor density

**Fig 1:**
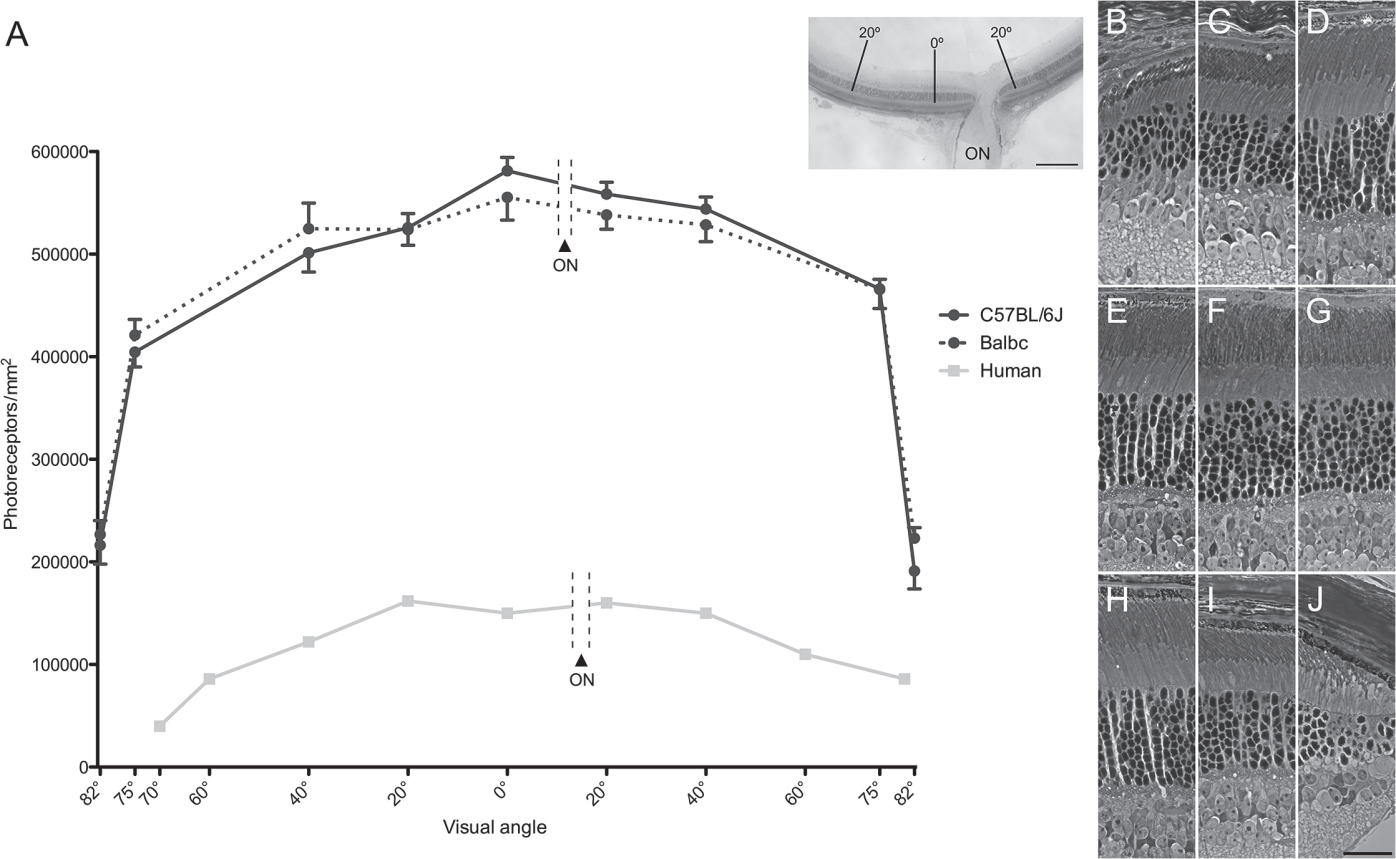
Comparison of photoreceptor distribution in mouse and human retinas. (A) Graph showing the photoreceptor density per mm^2^ in mouse and human. Data from the visual angles of 0°, 20° and 40° were collected at distances of 0, 0.6 and 1.2 mm, respectively, from the center of the mouse retina, along the dorso-ventral axis (shown left to right; ON indicates the location of the optic nerve head, which is just ventral from the center). Data from the visual angles of 75° and 82° were collected from regions centered at distances of 250 and 50 μm from the ora serrata, which approximated to 2.3 and 2.5 mm from the center. Error bars indicate SEM. Inset shows a low power micrograph of a dorso-ventral section passing through the optic nerve head and the center of the retina (0°); scale bar = 0.3 mm. The human data are from temporal to nasal, as reported by Osterberg [38]. Visual angles of 20°, 40°, 60° and 70° correspond to distances of 6, 12, 18 and 20 mm from the fovea. (B-J) Representative light microscopic images of the regions sampled along the dorsoventral axis of the mouse retinas. Examples from both the BALB/C and C57BL/6J strains are included. (B) 82° dorsal, (C) 75° dorsal, (D) 40° dorsal, (E) 20° dorsal, (F) center, (G) 20° ventral, (H) 40° ventral, (I) 75° ventral, (J) 82° ventral. Scale bar = 25 μm.

Rod and cone photoreceptor cell nuclei were counted at locations along the dorso-ventral axis of mouse retinas. These locations were specified based on their distances from the center of the retina, and their corresponding angle from the optical axis. The center was identified by the peak in photoreceptor cell density, and was thus found at a distance of 400 μm dorsal from the center of the optic nerve head (Fig 1A). This point coincides with that reported to lie on the optical axis of the mouse eye [35]. Cone nuclei were distinguished from rod nuclei by their distal location and chromatin distribution [36, 37].

Rod photoreceptor cell density, as determined from the counts of nuclei in both C57BL/6J and BALB/C mice, was observed to increase gradually from near the periphery to the center. At 75° off-axis, 1065 cells/2500 μm^2^ were counted (i.e. 426,000 cells/mm^2^), and, in the center, 1410 cells/2500 μm^2^ were counted (i.e. 564,000 cells/mm^2^), indicating a density that is 33% higher. Cone photoreceptor cell density, though much lower overall, changes in a similar manner across the retina. At 75° off-axis, 24 cone cells/2500 μm^2^ were counted (i.e. 9,600 cells/mm^2^), and, in the center, 32 cells/2500 μm^2^ were counted (i.e. 12,800 cells/mm^2^). Beyond 75° off-axis to the edge of the retina, at the ora serrata, photoreceptor cell density declines steeply (Fig 1; S1 Table).

Fig 1A shows a quantitative comparison between mouse and human retinas. Both the human and mouse retinas possess a higher photoreceptor cell density in the center than in the periphery. However, photoreceptor cell density is much higher throughout the mouse retina; in the central retina, it is 3–4 times that of the human macula.

Table 1 compares the rod and cone photoreceptor cell densities in the human macula and the central mouse retina of equivalent eccentricity in more detail. A striking characteristic of the human retina is the sharp peak of cone cell density present at 0°, which represents the small central region of the fovea known as the foveola. Such a cone cell peak is absent from the mouse retina. However, between 10° and 20°, representing the more peripheral part of the macula in the human retina, the cone cell density is higher in the mouse retina, and, interestingly, the rod/cone ratio is similar in human and mouse over most of this region.

**Table 1:** Mean rod and cone densities in different regions of the human macula and the central mouse retina.

Eccentricity	Rods/mm^2^ (x1000)		Cones/mm^2^ (x1000)		Rods/Cones	
	Human ^(1)^	Mouse ^(2)^	Human ^(1)^	Mouse ^(2)^	Human ^(1)^	Mouse ^(2)^
**0°**	0	564	150	13	0	44
**5°**	92	500	37	16	3	31
**10°**	116	515	8	16	15	33
**15°**	146	497	5	17	27	29
**20°**	149	507	5	15	30	34

^(1)^ Human data from [38] temporal from fovea

^(2)^ Mouse data from C57BL/6J; dorsal from optical axis

### RPE cells

**Fig 2:**
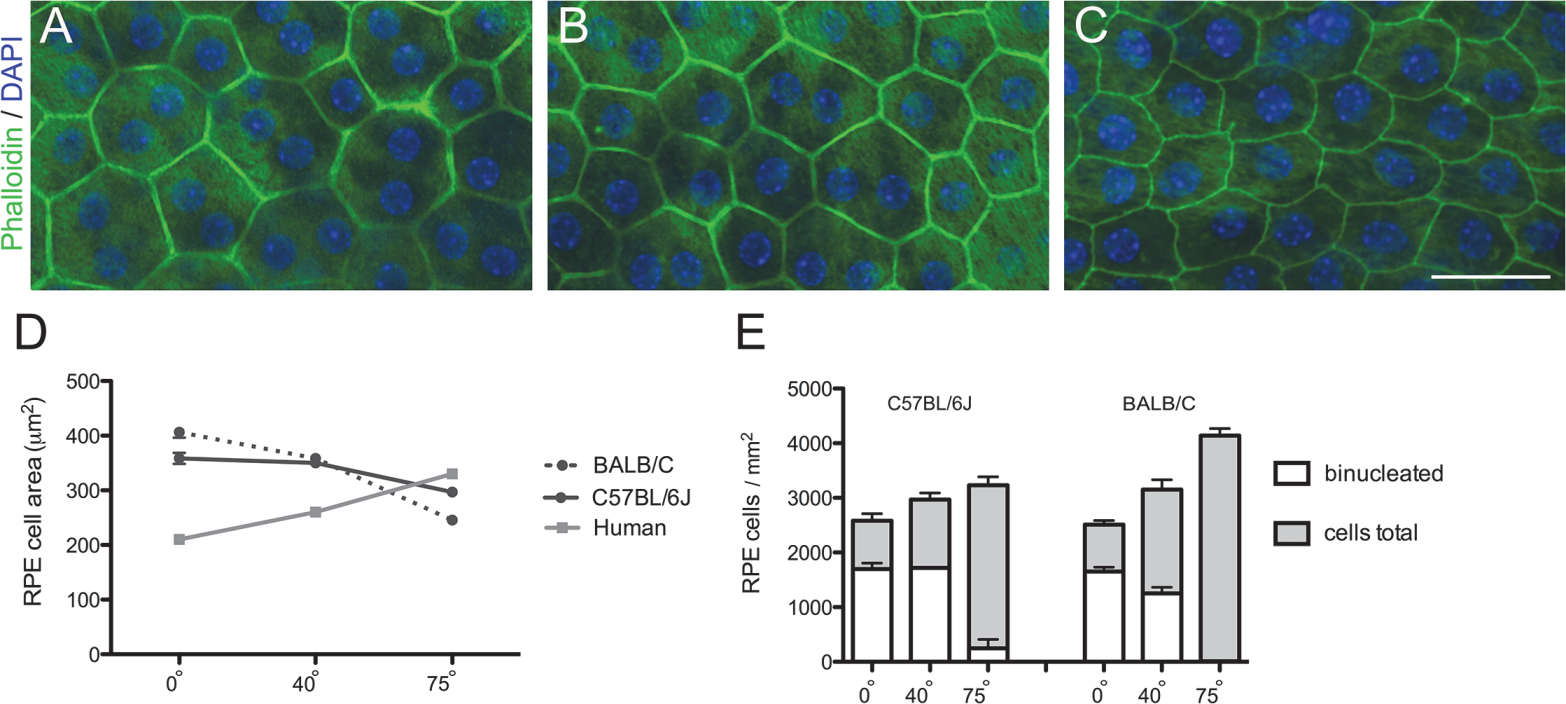
RPE cell size and relative abundance of binucleate RPE cells across the mouse and human retina. (A-C) Confocal images of a flatmount of a BALB/C mouse RPE, showing cells at retinal eccentricities of 0° (A), 40° (B), and 75° (C). Scale bar = 50 μm. (D) Graph of RPE cell cross-sectional area in relation to eccentricity in the mouse and human retinas. RPE cell size was significantly different between eccentricities in both mouse strains (*P* < 0.0001; Tukey’s test). (E) Graph of RPE cell density in relation to eccentricity in the mouse retinas, illustrating the proportion of binucleate cells, which was different between eccentricities in both mouse strains (*P* < 0.0001; Tukey’s test). Error bars in D and E indicate SEM. The human data are from Ts’o and Friedman [39].

RPE wholemounts were prepared from 5 mice of each strain, and the central, mid-peripheral, and peripheral regions of the retina, corresponding approximately to visual angles of 0°, 40°, and 75°, respectively, were imaged (Fig 2A–2C) and measured. A clear difference was observed in the cross-sectional areas between the central and peripheral RPE cells. RPE cell area was 1.7 times larger in the central area than in the periphery of BALB/C mice, while the difference in C57BL/6J mice was somewhat smaller (Fig 2D). In contrast, human data show the opposite trend [39], where RPE cells have a significantly smaller cross-sectional area in the center of the retina than in the periphery (Fig 2D). The apical to basal depth of the RPE is fairly constant across both the mouse and human [39] retinas at ~5 and ~7 μm, respectively.

As an aside, we also determined the number of binucleate RPE cells. We found that in the central retina of both mouse strains about 2/3rds of the RPE cells were binucleate, while in the peripheral region nearly all RPE cells had only one nucleus (Fig 2E). Overall, 35% of mouse RPE cells were binucleate, in contrast to only 3% in the human RPE [39].

### Photoreceptor to RPE cell ratio

**Fig 3:**
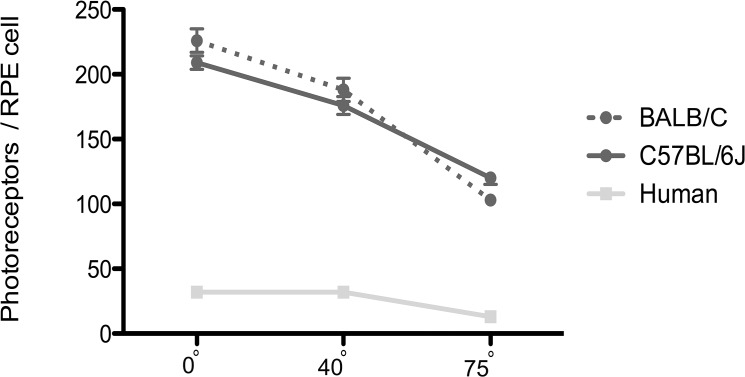
The number of photoreceptors per RPE cell in mouse and human eyes. This cell ratio differed at different eccentricities in both mouse strains (*P* < 0.0001; Tukey’s test). The human data are from Osterberg [38] and Ts’o and Friedman [39].

The number of photoreceptor cells per RPE cell in the center of the mouse retina is ~2-fold higher than that near the periphery (Fig 3). The difference in this ratio is due to both a higher photoreceptor cell density and a larger RPE cell size in the center. A comparable difference in this ratio is evident in the human retina, although this is due to a much sharper increase in photoreceptor cell density, which counters a smaller RPE cell size, in the macula (adapted from [38, 39]). The photoreceptor to RPE cell ratio is ~7-fold higher in the central and peripheral regions of the mouse retina when compared with these regions in the human retina.

### Bruch’s membrane

**Fig 4:**
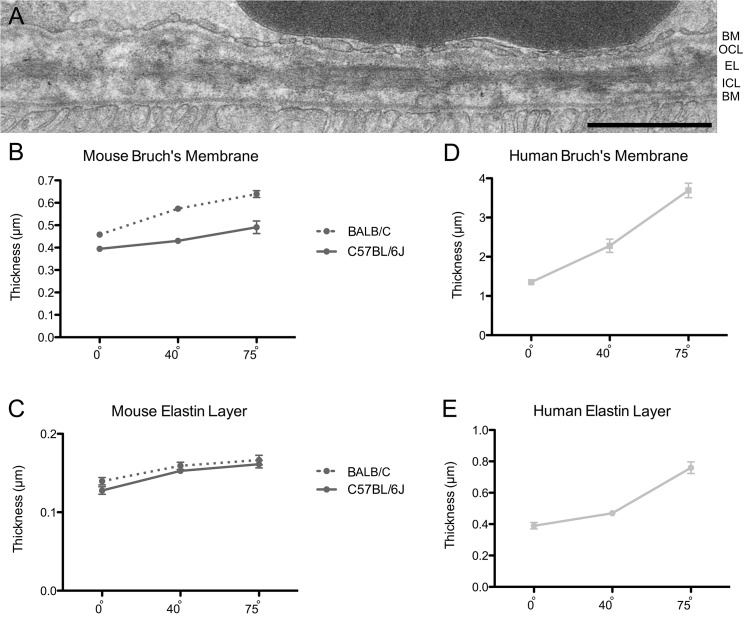
Bruch’s membrane and elastin layer thickness. (A) EM micrograph of Bruch’s membrane in a BALB/C mouse retina at an eccentricity of 40°. BM, Basement membrane; OC, Outer collagenous layer; EL, Elastin layer; ICL, Inner collagenous layer. Scale bar = 1 μm. (B, D) Graphs of the thickness of the entire Bruch’s membrane in relation to eccentricity in mouse and human retinas. (C, E) Graphs of the thickness of the elastin layer of Bruch’s membrane in relation to eccentricity in mouse and human retinas. The thickness of Bruch's membrane as well as the elastin layer varied very significantly between retinal eccentricities in both mouse strains (*P* < 0.0001; Tukey’s test). Error bars indicate SEM. The human data, shown in D and E, are from Newsome et al. [18].

To determine the thickness of the Bruch’s membrane in different regions of the retina, ultrathin sections of the central, middle and peripheral areas of the dorsal retina (visual angles of 0°, 40° and 75°) were examined by EM (Fig 4A). Overall, the BALB/C retina has a somewhat thicker Bruch’s membrane than the C57BL/6J retina. However, in the retinas of both mouse strains, Bruch’s membrane was found to be thinner in center than in the periphery (30–40% thicker) (Fig 4B). The elastin layer of Bruch’s membrane was also slightly thinner in the center than in the periphery (~20% thicker) (Fig 4C).

Available data show that Bruch’s membrane in the human retina tends to thicken with age. However, by comparing measurements from 10 to 45 year old donor eyes, as reported by Newsome et al. [18], with our mouse data, it is evident that the human Bruch’s membrane is thicker overall, by about 3 to 6 fold. Notably, as in the mouse retinas, the human Bruch’s membrane is thinner in the central area of the retina than in the periphery (Fig 4D). However the difference between center and periphery is more pronounced in the human retina; it is 2-fold thicker in the periphery [18]. Likewise, the magnitude of the center to periphery gradient in the thickness of the elastin layer is also somewhat greater in the human [17, 18] (Fig 4E).

Tabulation of mean RPE cell size, density, photoreceptor RPE cell ratio, and Bruch’s membrane and elastin layer thickness, at different eccentricities of the human and the mouse retina, is shown in S2 Table.

## Discussion

The present study has addressed the question of how the central retina of the mouse compares with the human macula, in the context of some structural specializations that may make the macula more susceptible to degeneration. The specializations are photoreceptor density, phagocytic load on the RPE, and the relative thinness of Bruch’s membrane. This question is important when considering the relevance of mouse models of macular degeneration, including AMD.

### Photoreceptor density

The most obvious specialization of the macula is the sharp increase in photoreceptor cell density. Here, we used data from Osterberg [38], who made measurements from one eye of a 16-year-old male. Since Osterberg [38], studies have reported a range of maximal foveal cone and perifoveal rod densities [11, 40–43], due to differences among individual donors as well as differences in methods of measurement [11, 42]. In a highly detailed study, Curcio et al. [11] reported a large range for maximal foveal cone density, from 120,000 to 324,000 cones/mm^2^, among different eyes. The density reported by Osterberg [38] (150,000 cones//mm^2^) lies within this range, although it is towards the low end.

The high density of photoreceptor cells in the human macula supports high spatial resolution. Interestingly, however, a high photoreceptor density is not a unique characteristic of retinas with a macula or fovea, since species that possess neither have comparable or even higher photoreceptor densities. Recently, it was reported that the maximal density of cone photoreceptors in the area centralis of the canine retina compares to that of the human fovea [44]. While many other animals lack such a high density of cone photoreceptors, a number are known to have rods that exceed this density. In the center of the domestic cat retina [45], for example, rod photoreceptors are packed more than twice as densely, at ~460,000/mm^2^. The present data on the mouse retina (Fig 1A; Table 1), which are consistent with values reported by Carter-Dawson and LaVail [36] and Jeon et al. [46], show that the central mouse retina has a higher concentration of photoreceptors than even the cat. Therefore, although the macula is characterized by a high photoreceptor density, the central mouse retina actually surpasses the human macula in this respect.

A common criticism of the mouse as a model for macular degeneration is that it does not have a sufficient density of cones. Such a statement is true when comparing the mouse central retina with the center of the macula, but, as the present report illustrates, it is not true at eccentricities beyond 10°, which includes a large amount of the region still regarded as the macula in the human retina. Between 10° and 20°, the mouse retina contains a higher concentration of cones, and the rod/cone ratio is comparable between mouse and human (Table 1).

### Phagocytic load on RPE

A higher photoreceptor density will place a greater phagocytic load on the RPE, given a comparable rate of disk renewal per photoreceptor cell. RPE cells that have more disk membrane phagosomes to degrade are likely to be more susceptible to inefficiencies in this process, which have been suggested to result in the accumulation of undigested material [21, 47–49]. RPE pathology is a component of macular degeneration [15, 16], and, in most cases, appears to follow the accumulation of lipofuscin [25–29] and sub-RPE deposits, including drusen [30–33]. The rod and cone outer segments of the central mouse retina [36] and the human macula [38, 50–54] are all within 1–2 μm in diameter, although the mouse outer segments, which are ~20 μm long [36], are only about half the length of those in the human macula [52, 55]. The time taken for complete renewal of rod outer segments in mice is 10 days [56]. In the rhesus monkey, whose macular outer segments have similar dimensions to those in human, the complete renewal time is comparable at ~12 days [57]. Therefore, the phagocytic load from each photoreceptor cell in the human macula is probably about twice as high as that in the central mouse retina. However, due to a 3–4 fold higher photoreceptor density, the load on the RPE in the mouse is nearly twice as much as that in the human macula. The central mouse retina also has much larger RPE cells than the human macula, resulting in a phagocytic load per RPE cell that is more than 3-fold higher.

Thus, due to its relatively high phagocytic load, the mouse retina may be a sensitive model for analyses of perturbations in phagosome degradation in relation to macular degeneration. A transgenic mouse, with reduced activity of cathepsin D and impaired outer segment phagosome proteolysis, has been shown to possess basal deposits [6]. Similarly, mice, in which delayed phagosome degradation appears to be due to defective transport of phagosomes and/or lysosomes within the RPE, develop AMD-like pathogenesis, including basal laminar deposits, oxidative stress and inflammation [58, 59].

### Bruch’s Membrane

Data from Newsome et al [18] show that Bruch’s membrane of the human retina is thinner in the macula. Chong et al. [17] focused on the elastin layer, which is sandwiched between the inner and outer collagen-rich layers (Fig 4A). They found that the elastin layer in the macula is substantially thinner and more porous than in other regions of the retina. From their results, they proposed that this regional difference in Bruch’s membrane might contribute to the predilection of the macula to degeneration, following particular insults. The central thinning of Bruch’s membrane is not a general characteristic among different species; in the sheep retina, for example, there is a converse gradient, with a thicker Bruch’s membrane in the center than in the periphery [60]. As shown here, however, the mouse retina is closer to the human pattern, with a thinner Bruch’s membrane in the central retina. Nevertheless, this central to peripheral gradient is steeper in the human retina, which, in absolute terms, is also substantially thicker.

One of the most important functions of Bruch’s membrane is its involvement in the movement of molecules between the choroid and the RPE, including oxidized lipids, oxidized cholesterol and other waste products cleared by the RPE [61]. The thinness and porosity of Bruch’s membrane is important for this function. On the other hand, a thinner and more porous region of the membrane seems more likely to be susceptible to invasion by neovascularization from the choroid [17].

### Mouse as a model for macular degeneration

As mentioned at the outset, there are many characteristics that might make the macula more susceptible to macular degeneration. For example, as noted by Pikuleva and Curcio [62], center to periphery gradients in the human retina of esterified cholesterol [63] and blood flow in the choriocapillaries [64, 65] result in potentially important differences between the macula and the periphery. At present, it is unclear whether the mouse retina also possesses such characteristics.

Here we have focused on high photoreceptor density [38], resulting in high phagocytic load for the RPE, and thinness of Bruch’s membrane [17]. As in the human retina, these characteristics are increased in the mouse central retina relative to the periphery, but, quantitatively, the center-peripheral difference is markedly less in the mouse. A center-peripheral difference is important with respect to whether lesions might be more severe in the central retina, as they are in human macular degeneration. However, a more fundamental question is whether a mouse retina would be expected to manifest macular degeneration-like pathology at all.

As discussed above, our measurements would support the mouse as a sensitive model for perturbations that were promoted by especially a high photoreceptor cell density (and thus phagocytic load for the RPE). Moreover, at eccentricities that correspond to the more peripheral part of the macula in the human retina, the mouse retina contains a substantially higher density of cone photoreceptors, as well as rod photoreceptors. In this region, the rod to cone ratio is comparable between the human and mouse retinas. It is noteworthy that the peripheral parts of the macula are included in pathogenesis of macular degenerations, and rods typically degenerate before the cones [66, 67].

In conclusion, we have illustrated that a large central area of the mouse retina possesses some of the structural characteristics, which, in the human retina, have been suggested to make the macula more susceptible to degeneration.

## Supporting Information

S1 Table Mean rod, cone and overall photoreceptor densities at different eccentricities of the mouse retina. ^(1)^ along dorso-ventral axis.
(DOCX)

S2 Table Comparison of RPE cell size, density, photoreceptor RPE cell ratio, Bruch’s membrane and elastin layer thickness at different eccentricities of the human and the mouse retina. ^(1)^ Human data from [39]. ^(2)^ Human data from [38]. ^(3)^ Data for ratio derived from photoreceptor density and RPE cell area. ^(4)^ Human data from [18].
(DOCX)

S1 Raw Data Spreadsheet including all counts, measurements and calculations made. (XLSX)
