# Supplementary material for: A Comparison of Some Organizational Characteristics of the Mouse Central Retina and the Human Macula
Source: PLoS One. 2015 Apr 29;10(4):e0125631. doi: 10.1371/journal.pone.0125631 (PMC4414478; doi:10.1371/journal.pone.0125631)
Supplement: S1 Table — (1) along dorso-ventral axis. (DOCX) [file pone.0125631.s001.docx]

S1 Table: Mean rod, cone and overall photoreceptor densities at different eccentricities of the mouse retina.

| Eccentricity^(1)^ | Rods/mm^2^  (x1000) | | | | Cones/mm^2^ (x1000) | | | | Total Photoreceptors | |
| --- | --- | --- | --- | --- | --- | --- | --- | --- | --- | --- |
|  | C57BL/6J | | BALB/C | | C57BL/6J | | BALB/C | | C57BL/6J | BALB/C |
|  | Mean | SEM | Mean | SEM | Mean | SEM | Mean | SEM |  |  |
| **82°** | 210 | 18 | 223 | 12 | 6 | 1.9 | 4 | 1.7 | 216 | 227 |
| **75°** | 395 | 14 | 414 | 14 | 9 | 2.4 | 7 | 0.9 | 404 | 421 |
| **40°** | 488 | 18 | 518 | 22 | 14 | 1.7 | 7 | 2.2 | 502 | 525 |
| **20°** | 507 | 15 | 516 | 14 | 15 | 1.6 | 8 | 1.3 | 522 | 524 |
| **0°** | 564 | 12 | 543 | 21 | 13 | 1.5 | 12 | 1.4 | 577 | 555 |
| **20°** | 545 | 12 | 528 | 13 | 13 | 1.8 | 10 | 1.3 | 559 | 538 |
| **40°** | 531 | 11 | 517 | 15 | 13 | 2.5 | 11 | 2.3 | 544 | 528 |
| **75°** | 456 | 19 | 457 | 9 | 10 | 1.4 | 9 | 0.9 | 466 | 466 |
| **82°** | 184 | 17 | 219 | 9 | 7 | 1.6 | 5 | 1.7 | 191 | 224 |

^(1)^ along dorso-ventral axis
