# Supplementary material for: A Comparison of Some Organizational Characteristics of the Mouse Central Retina and the Human Macula
Source: PLoS One. 2015 Apr 29;10(4):e0125631. doi: 10.1371/journal.pone.0125631 (PMC4414478; doi:10.1371/journal.pone.0125631)
Supplement: S2 Table — (1) Human data from [39]. (2) Human data from [38]. (3) Data for ratio derived from photoreceptor density and RPE cell area. (4) Human data from [18]. (DOCX) [file pone.0125631.s002.docx]

S2 Table: Comparison of RPE cell size, density, photoreceptor RPE cell ratio, Bruch’s membrane and elastin layer thickness at different eccentricities of the human and the mouse retina.

| Eccentricity |  | **0°** | | **40°** | | **75°** | |
| --- | --- | --- | --- | --- | --- | --- | --- |
|  |  | Mean | SEM | Mean | SEM | Mean | SEM |
| RPE cell area (μm^2^) | Human^(1)^ | 210 | - | 260 | - | 330 | - |
|  | C57BL/6J | 359 | 10 | 350 | 5 | 297 | 3 |
|  | BALB/C | 406 | 10 | 359 | 7 | 246 | 2 |
| RPE cells / mm^2^ | C57BL/6J | 2580 | 131 | 2970 | 119 | 3230 | 157 |
|  | BALB/C | 2510 | 77 | 3150 | 183 | 4140 | 128 |
| Photoreceptors / RPE cell | Human^(2)^ | 32 | - | 32 | - | 13 | - |
|  | C57BL/6J^(3)^ | 207 | 5 | 176 | 7 | 120 | 5 |
|  | BALB/C^(3)^ | 226 | 9 | 188 | 9 | 103 | 4 |
| Bruch’s membrane thickness (μm) | Human^(4)^ | 1.35 | 0.33 | 2.28 | 0.16 | 3.69 | 0.18 |
|  | C57BL/6J | 0.38 | 0.01 | 0.43 | 0.01 | 0.49 | 0.02 |
|  | BALB/C | 0.46 | 0.01 | 0.57 | 0.01 | 0.64 | 0.02 |
| Elastin layer thickness (μm) | Human^(4)^ | 0.39 | - | 0.47 | - | 0.76 | - |
|  | C57BL/6J | 0.13 | 0.01 | 0.15 | 0.00 | 0.16 | 0.01 |
|  | BALB/C | 0.14 | 0.01 | 0.16 | 0.01 | 0.17 | 0.01 |

^(1)^ Human data from (Ts’o and Friedman, 1968)

^(2)^ Human data from (Osterberg, 1935; Ts’o and Friedman, 1968)

^(3)^ Data for ratio derived from photoreceptor density and RPE cell area

^(4)^ Human data from (Newsome et al., 1987)
